# Structures and Encapsulation Motifs of Functional Molecules Probed by Laser Spectroscopic and Theoretical Methods

**DOI:** 10.3390/s100403519

**Published:** 2010-04-08

**Authors:** Ryoji Kusaka, Yoshiya Inokuchi, Sotiris S. Xantheas, Takayuki Ebata

**Affiliations:** 1 Department of Chemistry, Graduate School of Science, Hiroshima University, Kagamiyama 1-3-1, Higashi-hiroshima, 739-8526, Japan; E-Mails: d096443@hiroshima-u.ac.jp (R.K.), y-inokuchi@hiroshima-u.ac.jp (Y.I.); 2 Chemical & Materials Sciences Division, Pacific Northwest National Laboratory, 906 Battelle Boulevard, P.O. Box 999, MS K1-83, Richland, WA 99352, USA; E-Mail: sotiris.xantheas@pnl.gov (S.X.)

**Keywords:** supersonic jet, laser spectroscopy, host-guest complex, crown ether, calixarene

## Abstract

We report laser spectroscopic and computational studies of host/guest hydration interactions between functional molecules (hosts) and water (guest) in supersonic jets. The examined hosts include dibenzo-18-crown-6-ether (DB18C6), benzo-18-crown-6-ether (B18C6) and calix[4]arene (C4A). The gaseous complexes between the functional molecular hosts and water are generated under jet-cooled conditions. Various laser spectroscopic methods are applied for these species: the electronic spectra are observed by laser-induced fluorescence (LIF), mass-selected resonance enhanced multiphoton ionization (REMPI) and ultraviolet-ultraviolet hole-burning (UV-UV HB) spectroscopy, whereas the vibrational spectra for each individual species are observed by infrared-ultraviolet double resonance (IR-UV DR) spectroscopy. The obained results are analyzed by first principles electronic structure calculations. We discuss the conformations of the host molecules, the structures of the complexes, and key interactions forming the specific complexes.

## 1. Introduction

**Figure 1.**
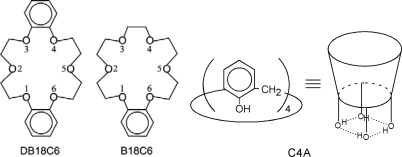
Schematic structures of dibenzo-18-crown-6-ether (DB18C6), benzo-18-crown-6-ether (B18C6), and calix[4]arene (C4A).

Crown ethers and calixarenes are known as functional molecules, which can encapsulate various neutral and ionic species within their cavities. Crown ethers are cyclic ethers built with several oxyethylene (-C-C-O-) units, whereas calixarenes are cyclic oligomers built with phenol units (Figure 1). They act as host molecules to encapsulate guest species in their cavities through non-covalent interactions, such as hydrogen(H)-bond and/or van der Waals (vdW) forces. Applications of crown ethers and calixarenes as molecular receptors, metal cation extraction agents, fluoroionophores and phase transfer catalytic media have been previously described in a number of studies in the literature [1–5].

One of the important aspects of these host/guest molecular systems is their selectivity in the encapsulation of guest species. There are two important factors controlling the selectivity: the size and the flexibility of the host cavity. If the size of the cavity of the host molecule matches that of the guest species, the host shows an efficient selectivity for the encapsulation of the particular species. For example, the 18-crown-6-ether (18C6) forms an exceptionally stable 1:1 complex with K^+^ [6,7] (compared to other alkali metal cations) because 18C6 forms a ring conformation of *D*_3d_ symmetry and the size of its cavity is comparable to the size of the spherical K^+^. As regards to calixarenes, *p-tert*-butylcalix[8]arene was found to selectively extract C_60_ from the mixture containing C_60_ and C_70_ [8,9].

Another important point for the selectivity is the effect of solvent molecules. In crown ethers, the preferential capture of K^+^ by 18C6 occurs in aqueous solution [10–15], while in the gas phase, 18C6 as well as 12-crown-4 (12C4) and 15-crown-5 (15C5) shows the largest binding energy to Li^+^ (not with K^+^) among the alkali-metal cations [16–20]. Previous studies suggested that the water solvation to the complexes enhances the binding energy with K^+^ [21,22]. Therefore, a stepwise study starting from the isolated molecule to micro-solvated complexes is essential to understand the mechanism of the encapsulation of crown ethers. Molecular clusters provide an ideal environment for the precise study of the micro-solvated effects under solvent-controlled conditions. Recently, such a study has been reported by Lisy and coworkers. [23–25]. They investigated the structure of the 18C6-alkali metal cation (LI^+^, Na^+^, K^+^, Rb^+^, Cs^+^, and Mn^2+^) complexes with solvent molecules (water and methanol) by IRPD spectroscopy and mass spectrometric techniques. Detailed investigation of the structure for the isolated and water-solvated crown ethers has been also carried out by Zwier and coworkers. [26–28]. They reported that “buckled” conformation of bare benzo-15-crown-5 (B15C) and 4′-aminobenzo-15-crown-5 (ABC) molecule(s) changes to an open conformation in the presence of water molecule(s) [27,28], which is a very similar result with our conclusions during studies of benzo-18-crown-6-ether/water system.

Here we review our studies on the structures of dibenzo-18-crown-6-ether (DB18C6), benzo-18-crown-6-ether (B18C6), calix[4]arene (C4A) and their complexes with water molecules [29–34]. The ultraviolet (UV) spectra of the jet-cooled bare molecules and corresponding complexes have been measured using laser-induced fluorescence (LIF), resonance enhanced two-photon ionization (R2PI) and UV-UV hole-burning (HB) spectroscopy. The infrared (IR) spectra of each species were measured by IR-UV double-resonance (DR) spectroscopy. The binding energy of the C4A-(H_2_O)_1_ complex was obtained by IR photodissociation (IRPD) spectroscopy. The electronic transition energies and the IR spectra are compared with those of the optimized structures obtained by quantum chemical calculations and discussed to determine the conformation and structure of the water containing complexes.

We first report on the structure of DB18C6, B18C6 and their hydrated complexes. Though the crown ethers are flexible, the substitution of benzene ring(s) to the crown frame makes it more rigid because the –O–C = C–O– (where C = C represents the carbon atoms in the benzene ring) frame prefers a planar structure. For this reason, B18C6 is more flexible than DB18C6 due to the smaller number of benzene rings in the crown frame and this may lead to the larger number of conformers under supersonic conditions. In addition, it is quite possible that the complexation with water alters the structures and relative energetics of the original conformations. We therefore investigate how the structural flexibility affects the dynamics of the encapsulation process at the microscopic level. We then study the structure of the hydrated complex of C4A. C4A has multiple hydroxyl groups connected by strong homodromic intramolecular hydrogen bonds in the lower rim of the cavity. The difference of the interaction of the guest species with either the benzene or the hydroxyl group sites is thought to be very subtle. Using a single water molecule as a guest, we investigate its preferential binding to either of those binding sites: inside the C4A cavity forming an *endo*-complex or outside the cavity forming an *exo*-complex. We address this fundamental question by combining laser spectroscopy and high-level quantum chemical calculations.

## 2. Approach

**Figure 2.**
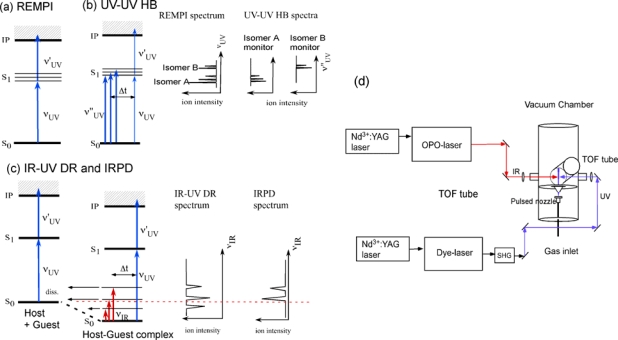
(a)–(c) Several laser spectroscopic methods with the ion signal detection. (d) Experimental setup for the supersonic beam and IR-UV DR spectroscopy.

***Experimental details***: Figure 2(d) shows the experimental setup of the supersonic beam and laser system. The supersonic jet of the functional molecules is generated by an adiabatic expansion of the gaseous mixture of the sample (host functional molecule) and water vapor into the vacuum chamber. The adiabatic expansion generates internally cold gaseous molecules and complexes, with most of them populated in the zero-point vibrational level. It should be noted that the cooling process in the expansion is the non-equilibrium process, so that several conformers at different local minima may coexist in the jet. To generate jet-cooled DB18C6, B18C6, C4A and their complexes with water molecules, we used a home-built high temperature pulsed nozzle. The pulsed nozzle consists of a commercially available valve and a sample housing made of polyimide resin. The sample housing that contains the sample powder is attached to the head of the valve and it is heated to ∼400 K to evaporate the nonvolatile sample. The housing has a 1 mm orifice at the exit. The poppet of the pulsed valve, which is also made of polyimide resin, is extended to the orifice of the sample housing and controls the injection of the sample gas. The gaseous mixture of the sample and water vapor, premixed with helium carrier gas at a total pressure of 2 bar, is expanded into the vacuum chamber through the orifice. A skimmer is used to generate a supersonic beam. The partial pressure of the water vapor is controlled by regulating the temperature of a water container connected to the gas lines.

We apply several laser spectroscopic methods for measuring the electronic and vibrational spectra of the species generated in the supersonic jet. For the measurement of the electronic spectrum we apply LIF and mass-resolved REMPI [Figure 2(a)] spectroscopy with nanosecond laser systems. The discrimination of the different species in the electronic spectra is carried out by UV-UV HB spectroscopy [Figure 2(b)]. In this technique two UV laser beams, namely the “pump” and “probe” beams, are used. The frequency of the probe UV laser is fixed to a band of a specific species and its fluorescence or ion signal is monitored. Under this setup the pump light is introduced prior (∼4 μs) to the probe one. When the pump laser frequency is resonant to a transition of the monitored species, these species is excited to the upper state resulting in the depletion of the fluorescence or the ion signal monitored by the probe light. The electronic spectrum of the monitored species is therefore obtained as the depletion spectrum as a function of the pump UV frequency. For the measurement of the IR spectrum of a specific complex we apply IR-UV DR spectroscopy [Figure 2(c)]. The principle of this technique is very similar to the UV-UV HB spectroscopy. Instead of the pump UV laser light we use a tunable IR laser to decrease the monitored signal. The IR laser is also introduced prior (∼100 ns) to the probe UV laser light and its wavelength is scanned. Depletion of the monitored signal occurs when the IR frequency is resonant to a vibrational transition of the monitored species and the IR spectrum in the S_0_ ground state is obtained as a depletion spectrum. For the C4A-(H_2_O)_1_ complex we used IR photodissociation (IRPD) spectroscopy for obtaining the binding energy [Figure 2(c)]. In that case the probe UV frequency is fixed to the electronic transition of C4A. When the absorbed IR energy is larger than the binding energy of the C4A-(H_2_O)_1_ complex, the complex dissociates to produce the C4A fragment. By scanning the IR frequency while monitoring C4A fragment we obtain the IRPD spectrum. The comparison between the IR-UV DR and IRPD spectra yields information about the binding energy of the complex. Figure 1(d) shows a typical setup of IR-UV double resonance spectroscopy.

***Theoretical calculations***: The optimum structures of DB18C6, B18C6 and their complexes with water were obtained by the density functional theory (DFT) calculation at the B3LYP/6-31+G* level with the GAUSSIAN 03 program package [35]. The energies of the optimized structures were corrected by zero-point vibrational energy. The harmonic vibrational frequencies were scaled by the factors of 0.9744 and 0.9524 for the OH and CH stretching vibrations, respectively, in order to compare with the experimentally measured ones. The S_1_–S_0_ electronic transition energies were calculated using time dependent density functional theory (TDDFT) at the B3LYP/6-31+G* level.

For C4A and the C4A-(H_2_O)_1_ complex the geometries were also optimized at the B3LYP/6-31+G** level but higher levels of electron correlation and larger basis sets were used in order to investigate the convergence of the results. These include the second order Moller-Plesset (MP2) level of theory [36] and the family of augmented correlation consistent basis sets of Dunning and co-workers [37,38] up to quadruple-zeta quality, aug-cc-pV*n*Z (*n* = D, T, Q). The MP2/aug-cc-pVDZ optimal geometries were used for single point calculations with the larger basis sets up to aug-cc-pVQZ. These calculations were performed with the NWChem suite of electronic structure codes [39] at Pacific Northwest National Laboratory. The MP2 binding energies are compared to the ones from DFT calculations at the B3LYP/6-31+G** level [40] which were obtained with the GAUSSIAN 03 program package. The harmonic vibrational frequencies were estimated at the MP2/aug-cc-pVDZ level of theory for the various isomers and scaled by 0.96 in order to produce the IR spectra that are compared to experiment.

The binding energy of the C4A-(H_2_O)_1_ complex is computed as:

(1) $$\Delta E(C4A-H_{2}O)=E_{C4A-H_{2}O}^{C4A-H_{2}O}(C4A-H_{2}O)-E_{C4A}^{C4A}(C4A)-E_{H_{2}O}^{H_{2}O}(H_{2}O),$$

where superscripts denote basis sets and subscripts indicate the geometries of the individual species identified in parentheses, viz. $E_{\mathrm{geometry}}^{\mathrm{basis set}}\left(\mathrm{species}\right)$. In this notation, for example, $E_{C4A-H_{2}O}^{C4A}\left(C4A\right)$ denotes the energy of C4A at the dimer [C4A-(H_2_O)] geometry with the monomer (C4A) basis set.

The basis set superposition error (BSSE) correction was estimated via the function counterpoise (fCP) method [41] including the fragment relaxation terms [42], which arise from the change in the intramolecular geometry of the C4A and H_2_O fragments in the complex minimum. Using the same notation introduced previously, the BSSE-corrected dimer binding energies are:

(2) $$\begin{array}{lll} \Delta E_{\mathrm{BSSE}}\;(C4A-H_{2}O) & = & E_{C4A-H_{2}O}^{C4A-H_{2}O}(C4A-H_{2}O)-E_{C4A-H_{2}O}^{C4A-H_{2}O}(C4A)-E_{C4A-H_{2}O}^{C4A-H_{2}O}(H_{2}O)\\ & + & E_{\mathrm{rel}}^{C4A}(C4A)+E_{\mathrm{rel}}^{H_{2}O}(H_{2}O) \end{array}$$

where

(3a) $$E_{\mathrm{rel}}^{C4A}(C4A)=E_{C4A-H_{2}O}^{C4A}(C4A)-E_{C4A}^{C4A}(C4A)$$

(3b) $$E_{\mathrm{rel}}^{H_{2}O}(H_{2}O)=E_{C4A-H_{2}O}^{H_{2}O}(H_{2}O)-E_{H_{2}O}^{H_{2}O}(H_{2}O)$$

are the fragment relaxation terms. Therefore 4 additional calculations (C4A and H_2_O with the full complex basis at the complex and at the isolated MP2/aug-cc-pVDZ geometries) are required for each BSSE calculation.

## 3. Results and Discussion

### 3.1. Dibenzo-18-Crown-6-Ether (DB18C6)

#### 3.1.1. Electronic Spectra of Jet Cooled DB18C6 and DB18C6-(H_2_O)_n_

**Figure 3.**
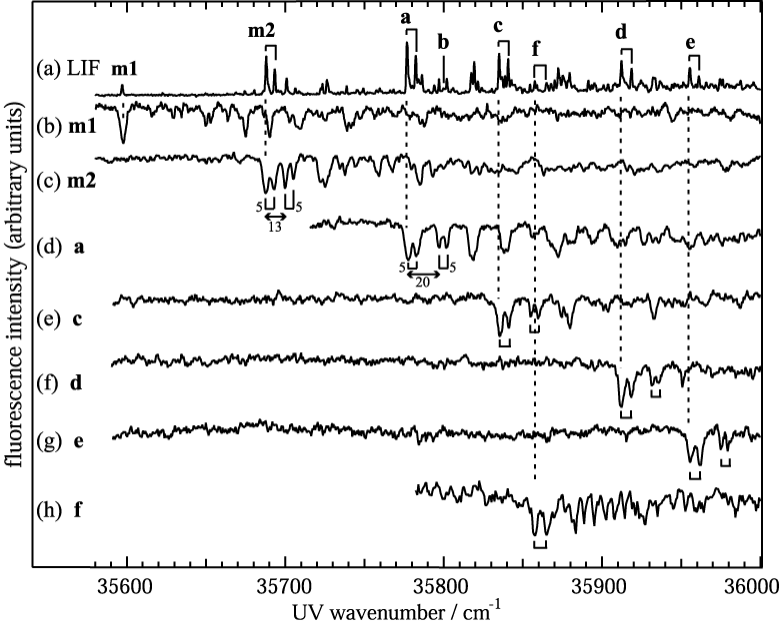
(a) LIF spectrum of jet-cooled DB18C6 and its hydrated complexes. (b)–(h) UV-UV HB spectra measured by monitoring bands **m1**, **m2**, **a**, and **c–f** in the LIF spectrum, respectively. The numbers in (c) and (d) show the energy interval (cm^−1^) in the corresponding regions. Figure adapted from Reference [28].

Figure 3(a) shows the LIF spectrum of the jet-cooled bare DB18C6 conformers and the DB18C6-(H_2_O)*_n_* complex indicating the S_1_-S_0_ transitions. Figures 3(b)–(h) show the UV-UV HB spectra obtained by monitoring bands **m1**, **m2**, **a**, and **c–f**, respectively. From these spectra, each of the **m1**, **m2**, **a**, and **c–f** transitions is ascribed to different species. The **m1** and **m2** transitions correspond to the bare DB18C6 conformers, since they do not exhibit any bands corresponding to the OH stretching vibrations of water molecule(s) in the IR-UV DR spectra. Transitions **a–f** are attributed to the DB18C6-(H_2_O)*_n_* complexes, because they do exhibit IR bands of OH stretching vibrations of water molecule(s), which will be described in later section. The positions of the origin bands are listed in Table 1.

#### 3.1.2. Conformation of DB18C6

**Figure 4.**
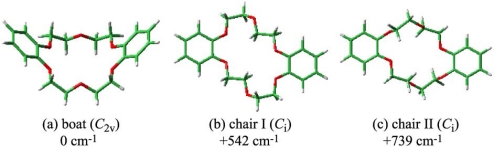
Optimized conformers of DB18C6: (a) boat, (b) chair-I, and (c) chair-II. The numbers shown in cm^−1^ represent the electronic energies relative to that of the most stable conformer (boat). Figure adapted from Reference [28].

We first discuss the conformation of the bare DB18C6 corresponding to species **m1** and **m2**. Figure 4 shows the three most stable conformers of DB18C6 optimized at the B3LYP/6-31+G* level of theory. We will refer to the most stable conformer as the “boat” and the other two higher lying conformers as “chair-I” and “chair-II”, respectively. In the chair-I conformer all atoms of the –C–O–C=C–O–C– frames lie on the same plane, whereas in the chair-II conformer two of the four –O–CH_2_– frames are twisted out of the plane. Table 2 lists the S_1_-S_0_ transition energies of the three conformers obtained by a TDDFT calculation at the B3LYP/6-31+G* level. The transition energy of chair-I is the lowest among the three conformers, while that of chair-II is the highest. The difference of the transition energies is attributed to the difference of the π-orbital delocalization on the benzene rings. The electronic transition of DB18C6 is due to the π-π* transition of the phenyl groups, such as *o*-dimethoxy benzene (DMB), and the π-π* transition energy decreases when the π-orbitals extend to the crown ether frame. The delocalization in the boat and chair-I isomers is larger than that in chair-II because all atoms of the –C–O–C=C–O–C– frame in the boat and chair-I isomers are on the same plane of the benzene ring, while in the chair-II isomer two of the four frames are twisted out of the plane of the benzene rings. The smaller degree of delocalization in chair-II results in a higher S_1_-S_0_ transition energy than the corresponding one for the boat and chair-I isomers. The relative stability in the S_0_ state and the S_1_-S_0_ transition energies of the three conformers suggest that **m2** can be assigned to the boat conformer and **m1** to the chair-I conformer. These assignments are consistent with the fact that both the boat and chair-I conformations were found in a crystal [43].

Further experimental support for the assignment of **m2** as the boat conformer is obtained by the band splitting of 5 cm^−1^ as shown in Figure 3(c). A possible origin of this splitting is due to exciton splitting in excited electronic states. DB18C6 can be regarded as the combination of two DMB moieties, so the excited electronic states of DB18C6 are expressed by linear combinations of $\phi_{A}^{*}\phi_{B}$ and $\phi_{A}\phi_{B}^{*}$ of the two constituent fragments:

(4a) $$S_{1}:\phi_{A}^{*}\phi_{B}+\phi_{A}\phi_{B}^{*}$$

(4b) $$S_{2}:\phi_{A}^{*}\phi_{B}-\phi_{A}\phi_{B}^{*},$$

where ϕ* and ϕ correspond to the S_1_ and S_0_ electronic states of DMB, respectively. For the boat conformer (*C*_2v_ symmetry) the transitions from the S_0_(*A*_1_) state to both the S_1_(*B*_1_) and S_2_(*A*_1_) states are dipole allowed. On the contrary, in the chair conformers (*C*_i_ symmetry) only the transition to the S_1_(*A*_u_) state is dipole allowed. Therefore the 5 cm^−1^ splitting of the **m2** band corresponds to the S_2_-S_1_ energy difference of the boat conformer, while this splitting is not observed for **m1**.

**Scheme 1.**
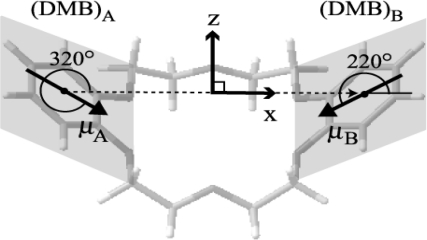
Symmetry axes and transition dipole moments of two DMB chromophores (*μ*_A_ and *μ*_B_). Figure adapted from Reference [28].

The magnitude of the exciton splitting can be estimated by the weak interaction model [44,45]. The S_2_-S_1_ splitting energy of a molecule having two equivalent chromophores is given by:

(5) $$\Delta E=2FV_{\text{AB}}$$

where:

(6) $$V_{\mathrm{AB}}=\frac{\mu_{A}\mu_{B}}{4\pi \varepsilon_{0}R_{\mathrm{AB}}^{3}}(2\text{cos}\theta_{A}\text{cos}\theta_{B}-\text{sin}\theta_{A}\text{sin}\theta_{B}\text{cos}\varphi )$$

is the dipole-dipole interaction energy between the two transition dipoles (*μ*_A_ and *μ*_B_), *R*_AB_ is the distance between the two centers of the chromophores, *θ*_A_ and *θ*_B_ are the angles of the transition dipoles to the line connecting the two centers and *φ* is the dihedral angle between the two transition dipoles (Scheme 1). *V*_AB_ and *F* denote the electronic and vibrational parts, respectively, contributing to the energy splitting Δ*E*. In order to obtain *V*_AB_ we first calculated the oscillator strength for the S_1_-S_0_ transition of DMB at the TDDFT B3LYP/6-31+G* level. The calculated S_1_-S_0_ oscillator strength of DMB is 0.049, which corresponds to a transition dipole moment of *μ*_DMB_ = 5.5 × 10^−30^ C·m. In the boat conformer the values for *θ*_A_, *θ*_B_, *φ* and *R*_AB_ are 320°, 220°, 0°, and 8.8 Å, respectively. Using these numbers we obtain a value of 70 cm^−1^ for 2*V*_AB_ from eq. (6). The estimation of 70 cm^−1^ for 2*V*_AB_ is further supported by the TDDFT result of 82 cm^−1^ for the boat conformer, as seen in Table 2. The value of *F*, corresponding to the Franck-Condon factor, is roughly estimated to be 0.1 from the UV-UV HB spectrum of species **m2**. Using these values we obtain an exciton splitting energy Δ*E* = 7 cm^−1^, which is in reasonable agreement with the experimental observation of 5 cm^−1^. Therefore the 5 cm^−1^ splitting for the species **m2** is attributed to the exciton splitting of the boat conformer of DB18C6.

#### 3.1.3. Hydrated Complexes of DB18C6

**Figure 5.**
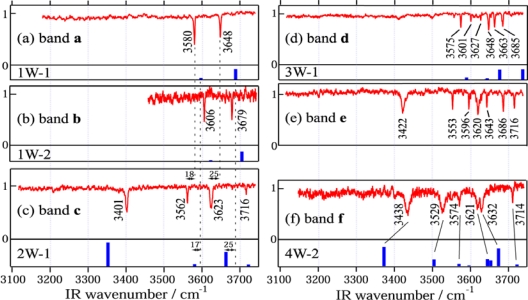
(a)–(f) IR-UV DR spectra of DB18C6-(H_2_O)*_n_* measured by monitoring bands **a–f** in the LIF spectrum, respectively. Sticks under the IR-UV DR spectra denote the calculated IR spectra at the optimized structures. Figure adapted from Reference [28].

All bands of the hydrated complexes of DB18C6 (bands **a**, **c–f**) exhibit a 5 cm^−1^ splitting as seen in Figure 3(d)–(h). This suggests that in the hydrated complexes DB18C6 has the boat conformation. The H-bonding network present in the hydrated complexes of DB18C6 is investigated by analyzing the IR-UV DR spectra in the OH stretching region, which are shown in Figure 5.

##### 3.1.3.1. DB18C6-(H_2_O)_1_

The structure of the IR spectrum in the OH stretching region (3100–3750 cm^−1^) corresponds to the “fingerprint” of the underlying H-bonding network. Figures 5(a) and (b) show the IR-UV DR spectra where bands **a** and **b** are monitored. Since the two bands appear at different positions in each spectra, species **a** and **b** are due to different isomers of the DB18C6-(H_2_O)_1_ complex. The intensity of band **a** in the LIF spectrum is more than 10 times larger than that of band **b**, suggesting that the former is the major species in the jet. The positions of the two vibrations for the species **a** and **b** are red shifted with respect to the vibrations of water [46] by 77 and 108 cm^−1^ (for band **a**) and by 51 and 77 cm^−1^ (for band **b**). This suggests that in both complexes the two OH groups of the water molecule are H-bonded, that is, the water molecule forms a bidentate H-bonded structure with the ether O atoms. Further structural information for the species corresponding to bands **a** and **b** is obtained from the blue shifts of 89 and 112 cm^−1^ of those bands in the LIF spectrum with respect to band **m2** of the DB18C6 boat isomer. In the DMB-(H_2_O)_1_ complex a water molecule is bound to the O atoms of the methoxy groups via two H-bonds and the origin band is blue-shifted by 127 cm^−1^ from the origin of bare DMB [47]. Based on the similarity of the blueshift to DMB-(H_2_O)_1_ case, species **a** and **b** can be probably assigned to structures in which a water molecule is H-bonded to the O atom(s) next to the benzene ring(s).

**Figure 6.**
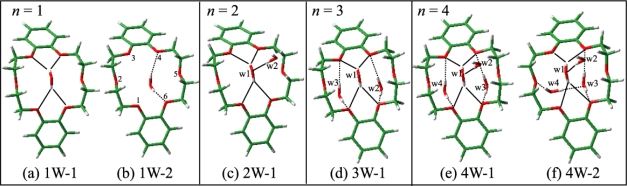
Optimized structures of DB18C6-(H_2_O)_1−4_, except for structure 4W-1 which represents a hypothetical probable structure. Solid lines denote H-bonds from the bottom side of the boat DB18C6, whereas dotted lines correspond to H-bonds from the top side. Figure adapted from Ref. [28].

**Figure 7.**
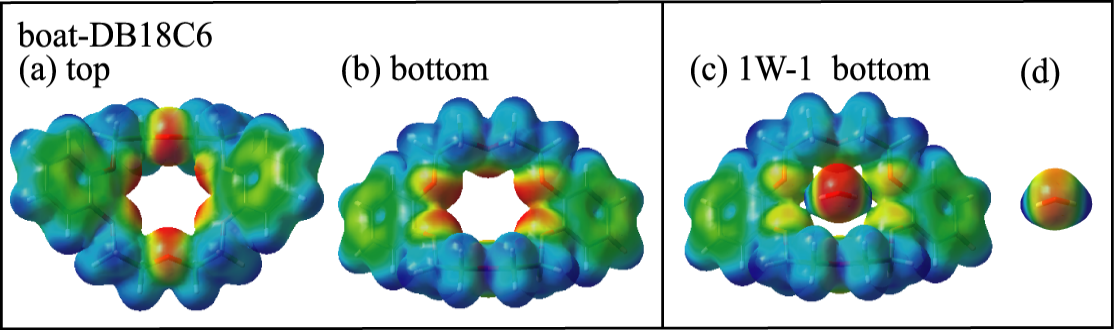
Electrostatic potential of DB18C6 from −0.08 a.u. (red) to +0.08 a.u. (blue) on the surface of the same electron density (0.01 $e/a_{0}^{3}$). Figure adapted from Reference [28].

The proposed structures for species **a** and **b** are supported by DFT calculations. Figures 6(a) and (b) show the optimized structures (1W-1 and 1W-2) of the DB18C6-(H_2_O)_1_ complex. In both structures the conformation of DB18C6 is the boat form and the water molecules are H-bonded to the O atoms next to the benzene rings by bidentate H-bonding. In 1W-1 each of two OH groups points to the middle positions between the O_1_ and O_6_ and between the O_3_ and O_4_ atoms, respectively. The H-bonding pattern in which one OH group is H-bonded to two ether O atoms corresponds to a “bifurcated” H-bond [48,49]. On the other hand, in the 1W-2 isomer the two OH groups are bonded directly to the O_4_ and O_6_ atoms, respectively. The calculated IR spectra for 1W-1 and 1W-2 are shown in Figures 5(a) and (b) as a stick diagram. Both the 1W-1 and 1W-2 spectra display the bidentate symmetric and anti-symmetric OH stretching vibrations around ∼3600 and ∼3700 cm^−1^, respectively. Species **a** and **b** can be assigned to 1W-1 and 1W-2, respectively, since the OH stretching frequencies of 1W-1 are slightly red-shifted with respect to those of 1W-2 and therefore are associated with stronger hydrogen bonds. The reason for the stronger H-bond in 1W-1 with respect to 1W-2 can be examined by the charge distribution on the O atoms in the DB18C6 boat conformer. Figures 7(a) and (b) display the top and bottom views of the electrostatic potential of the boat conformer. The negative charge on the O_1_, O_3_, O_4_ and O_6_ atoms is more exposed in the bottom than in the top. Therefore a water molecule in the 1W-1 isomer can form a stronger H-bond than in the 1W-2 isomer.

##### 3.1.3.2. DB18C6-(H_2_O)_2_

The IR-UV DR spectrum of species **c**, shown in Figure 5(c), displays four OH stretching bands, suggesting that species **c** corresponds to the DB18C6-(H_2_O)_2_ complex. The bands at 3562 and 3623 cm^−1^ are assigned to the symmetric and anti-symmetric OH stretching vibrations of a bidentate water molecule, respectively. Their positions are red-shifted by 18 and 25 cm^−1^ with respect to those of band **a**, respectively, suggesting the O atom of the bidentate water acts as an acceptor of the H-bond for the second water molecule. The bands at 3401 and 3716 cm^−1^ can be assigned to the singly H-bonded and free OH stretching vibrations of the second water molecule, respectively. Figure 6(c) shows the most probable structure for species **c** (2W-1). In 2W-1 the second water molecule (w2) is H-bonded to the O atom of the bidentate water molecule (w1). The calculated IR spectrum for 2W-1, shown in Figure 5(c), reproduces the experimentally measured IR-UV DR spectrum of species **c** quite well. One noticeable feature of the spectra of species **c** is that the singly H-bonded OH stretching frequency (3401 cm^−1^) is much lower than that of water molecules forming a normal H-bond. For example, the frequency of the donor OH stretching vibration in the water dimer is 3530 cm^−1^[50]. The strong H-bond in species **c** can be examined by the charge distribution in 1W-1. Figure 7(c) shows the bottom view of the electrostatic potential of 1W-1. The O atom of the bidentate water molecule is highly charged compared with that of a bare water molecule [Figure 7(d)]. Thus the first water molecule (w1) in 1W-1 is a good target for the second water molecule (w2) to form a H-bond.

##### 3.1.3.3. DB18C6-(H_2_O)_3_

The IR-UV DR spectrum of band **d** in Figure 5(d) exhibits six OH stretching bands, suggesting that species **d** can be assigned to the DB18C6-(H_2_O)_3_ complex. The difference with the IR spectrum of species **c** is that all the bands are located close to each other and no band appears at the free OH stretching region (∼3715 cm^−1^). Therefore all water molecules form bidentate H-bonds in species **d** and we can furthermore classify the six bands into three pairs: (i) 3575 and 3648 cm^−1^, (ii) 3601 and 3663 cm^−1^, and (iii) 3627 and 3685 cm^−1^. In each pair the lower and higher frequency bands can be assigned to the symmetric and anti-symmetric OH stretching vibrations of the bidentate water, respectively. The lowest pair of the frequencies (i) is attributed to a bidentate water molecule bound to the bottom of the boat conformer and the other two pairs [(ii) and (iii)] arise from the water molecules forming weaker bidentate H-bonds at the opposite (top) side of DB18C6. Figure 6(d) depicts the most probable structure of species **d** (3W-1). In this structure the first water molecule (w1) forms a bidentate H-bond in the bottom side of the boat DB18C6 isomer like in 1W-1 and the second (w2) and third (w3) water molecules form two bidentate H-bonds at the opposite side like in 1W-2. The calculated IR spectrum for 3W-1 is shown in Figure 5(d). Though each of the bands at ∼3650 and ∼3730 cm^−1^ appears to be a single band, there are in reality two bands at each position. The positions of the six bands of 3W-1 agree well with those of the IR-UV DR spectrum of species **d**.

##### 3.1.3.4. DB18C6-(H_2_O)_4_

The IR-UV DR spectrum of band **e** in Figure 5(e) shows seven bands and the band at 3620 cm^−1^ has a shoulder, indicating that species **e** is due to the DB18C6-(H_2_O)_4_ complex. The 3422 and 3716 cm^−1^ bands can be assigned to the H-bonded and free OH stretching vibrations of a single-donor water molecule, respectively. By comparing with that of species **d**, we realize that the structure of the IR bands in the 3550–3690 cm^−1^ region is very similar to species **d**. This suggests that the IR bands of species **e** can be assigned to the OH stretching bands of three water molecules H-bonded like those in 3W-1. Unfortunately, our DFT calculations were unable to yield an isomer of the DB18C6-(H_2_O)_4_ complex whose IR spectra match the observed IR-UV DR spectrum of species **e**. The structure shown in Figure 6(e) (4W-1) represents a hypothetical probable structure for species **e**.

The IR-UV DR spectrum of species **f** is shown in Figure 5(f). Similarly to species **c** and **e**, the 3,438 cm^−1^ band can be assigned to the single-donor OH stretching vibration and the band at 3714 cm^−1^ is assigned to the free OH stretching vibration. The 3529 cm^−1^ band is unique to species **f** since the other species do not show a band around 3530 cm^−1^. The 3529 cm^−1^ band is located on the lower frequency side of the bidentate symmetric OH stretching vibration. In addition, the width of the 3,529 cm^−1^ band is broader than those for the bidentate water molecules. Therefore the 3529 cm^−1^ band cannot be assigned to a bidentate water molecule that is H-bonded to the ether O atoms. It is therefore necessary to consider a different type of H-bond that gives rise to the 3529 cm^−1^ band. Figure 6(f) shows a probable structure for species **f** (4W-2). In 4W-2, the first and second water molecules (w1 and w2) construct a bidentate and single-donor H-bonded network like in 2W-I, whereas the third water molecule (w3) is bonded to the O_4_ and O_6_ atoms like in 1W-2 and the fourth water molecule (w4) forms a bridge between an ether O atom (O_2_) and the O atom of (w3). This type of H-bonding network was also found in the 18-crown-6-ether/water system at the liquid nitrogen temperature [51]. The calculated IR spectrum for 4W-2 is displayed in Figure 5(f). As traced by solid lines, the calculated IR spectrum for 4W-2 reproduces the structure of the measured IR-UV DR spectrum of species **f** quite well. In particular, the calculated IR spectrum predicts the band at ∼3,500 cm^−1^, which is the stretching vibration of (w4) bonded to the O atom of (w3). This band corresponds to the band at 3529 cm^−1^ in the measured IR-UV DR spectrum of species **f**.

### 3.2. Benzo-18-Crown-6-Ether (B18C6)

#### 3.2.1. Electronic Spectra of B18C6 and B18C6-(H_2_O)_n_

**Figure 8.**
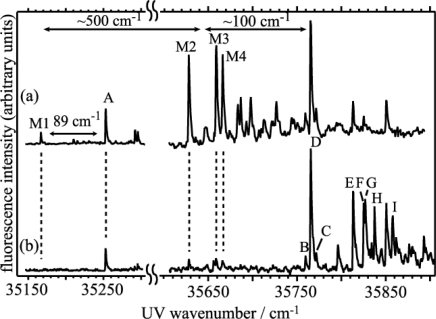
LIF spectra of jet-cooled B18C6 and its hydrated complexes obtained (a) without and (b) with adding water vapor. Figure adapted from Reference [29].

As was described in the introduction, B18C6 is more flexible than DB18C6. We investigate how the difference in flexibility affects its conformation as well as its complexation with water. Figure 8 shows LIF spectra of B18C6 in the band origin region; the spectra in Figure 8(a) and (b) were measured without and with adding water vapor, respectively. The addition of water vapor reduces the intensities of bands **M1–M4** and increases those of bands **A–I**. This result suggests that the bands **M1–M4** and **A–I** are due to bare B18C6 and the B18C6-(H_2_O)*_n_* complexes, respectively. From the UV-UV HB spectra obtained by monitoring each of the bands, though not shown here, each of the bands **M1–M4** and **A–I** are attributed to the bands of different species. The IR-UV DR spectra for bands **M1–M4** do not show any bands in the OH stretching region, so they are due to conformers of bare B18C6 (the number of the B18C6 conformers is larger than that for DB18C6 conformers). Band **A** and bands **B–D** are located at ∼100 cm^−1^ higher frequency than band **M1** and bands **M2–M4**, respectively. From the origin band positions of DB18C6-(H_2_O)_1_ and DMB-(H_2_O)_1_ with respect to the bare molecules the species **A** and the species **B–D** can be assigned to isomers of B18C6-(H_2_O)_1_ built on **M1** and **M2–M4**, respectively. Bands **E–I** show larger blue shifts, so that they are assigned to larger hydrated complexes of conformers **M2–M4**. Interestingly, the intensity of band **D** is much stronger than those of the other isomers of B18C6-(H_2_O)_1_, which means that species **D** is selectively generated among the B18C6-(H_2_O)_1_ isomers.

#### 3.2.2. IR Spectra in the OH Stretching Region and the H-Bonded Network

**Figure 9.**
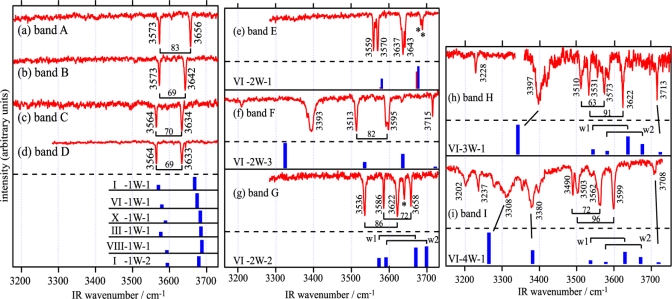
IR-UV DR spectra obtained by monitoring bands A-I in the LIF spectrum. Stick spectra represent calculated IR spectra at the optimized structures of B18C6-(H_2_O)_1−4_. In the calculated spectrum of VI-2W-1, two pairs of the bidentate OH stretching vibrations (red and blue sticks) are located close to each other. Figure adapted from Reference [29].

**Figure 10.**
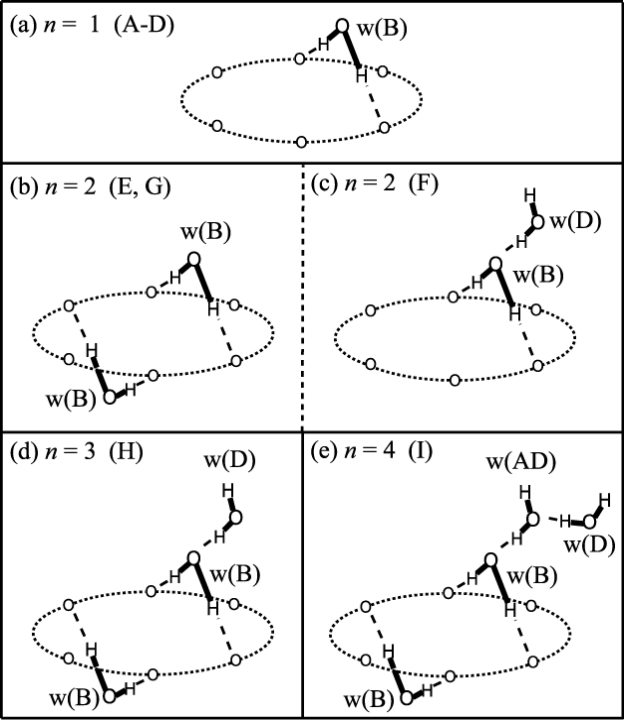
Geometric features deduced from the analysis of the IR-UV DR spectra in the OH stretching region of species A–I of the B18C6-(H_2_O)*_n_* complexes. w(B) and w(D) stand for bidentate and singly H-bonded H_2_O molecules, respectively. The H_2_O molecule labeled as w(AD) takes part in H-bonding as both acceptor and donor. Figure adapted from Reference [29].

The study on the DB18C6-(H_2_O)*_n_* complexes in the previous section makes the determination of H-bond networks in B18C6-(H_2_O)*_n_* more straightforward. The IR-UV DR spectra of bands **A–I** in Figure 9 can be easily understood by the schematic structures shown in Figure 10. Species **A–D** have one water molecule bidentately H-bonded as shown in Figure 10(a) because each of the IR-UV DR spectra of species **A–D** [Figure 9(a)–(d)] shows one pair of bidentate OH stretching vibrations.

The IR-UV DR spectra of species **E** and **G** [Figures 9(e) and (g)] show two pairs of bidentate OH stretching vibrations (at 3559, 3570, 3637, and 3643 cm^−1^ for species **E** and at 3536, 3586, 3622, and 3658 cm^−1^ for species **G**), so that species **E** and **G** have two water molecules bidentately H-bonded [Figure 10(b)]. As to the weak bands at 3684 and 3689 cm^−1^ labeled by asterisks in Figure 9(e) and the one at 3641 cm^−1^ in Figure 9(g), they are probably due to combination bands of intermolecular stretching and the symmetric OH stretching vibrations [52]. The IR-UV DR spectrum of species **F** shows one pair of bidentate OH stretching (at 3513 and 3595 cm^−1^), a singly H-bonded OH stretching (at 3393 cm^−1^) and a free OH stretching (at 3715 cm^−1^) vibrations, suggesting that the structure of H-bond network in species **F** is the structure shown in Figure 10(c).

The IR-UV DR spectrum of species **H** [Figure 9(h)] exhibits four bands in the 3500–3650 cm^−1^ region that are assigned to two pairs of the bidentate OH vibrations, a singly H-bonded OH stretching (at 3397 cm^−1^) and a free OH stretching (at 3713 cm^−1^) vibrations. The weak band at 3228 cm^−1^ can be assigned to the overtone of the bending vibration of H_2_O [53]. Thus, in species **H** two H_2_O molecules are bidentately H-bonded, and another H_2_O molecule is singly H-bonded to either of the two bidentate H_2_O molecules as shown in Figure 10(d). The IR-UV DR spectrum of species **I** [Figure 9(i)] exhibits two pairs of bidentate OH stretching vibrations in the 3450–3650 cm^−1^ region and the 3308 and 3380 cm^−1^ bands attributed to the singly H-bonded OH stretching vibrations. The existence of the two singly H-bonded OH bands suggests that water molecules form a H-bonding chain as denoted by w(AD) and w(D) in Figure 10(e).

#### 3.2.3. Conformation of B18C6

**Figure 11.**
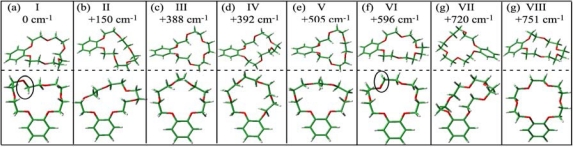
Optimized structures of conformers of B18C6 at the B3LYP/6-31+G* level. The top and side views are shown for each isomer. The numbers shown in cm^−1^ represent the electronic energies relative to that of the most stable structure (conformer I). Conformers I and VI are similar to each other; only the orientations of the O atoms highlighted by the solid circles in (a) and (f) are different. Figure adapted from Ref. [29].

Figure 11 shows the eight most stable conformers of bare B18C6 (I–VIII) obtained at the B3LYP/6-31+G* level of theory. It should be noted that the conformers I and VI are similar to each other; only the orientations of the O atoms highlighted by the solid circles in Figure 11(a) and (f) are different. We first tried to determine the conformations of the four species **M1–M4** from the IR spectra in the CH stretching region. However, the comparison between the IR-UV DR spectra of **M1–M4** (which are not shown here) and the calculated IR spectra of the conformers I–VIII was not adequate in order to allow us to be able to assign the structures of species **M1–M4**, though the IR-UV DR spectra of species **M1–M4** clearly show the different IR spectral patterns from each one. Instead we relied on the S_1_–S_0_ transition energy for the assignment of the conformation.

**Figure 12.**
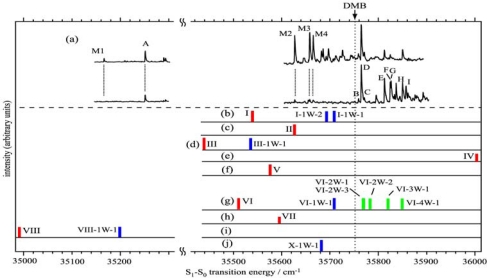
(a) LIF spectra of B18C6. (b–j) calculated S_1_-S_0_ electronic spectra for B18C6-(H_2_O)_0−4_ with the TDDFT method at the B3LYP/6-31+G* level. The red, blue, and green bars represent the electronic transitions of bare B18C6, B18C6-(H_2_O)_1_, and B18C6-(H_2_O)_2−4_, respectively. The electronic transitions of B18C6-(H_2_O)*_n_* having the same B18C6 conformation are drawn in the same row. The calculated spectra are scaled by 0.89599 so that the calculated transition energy of DMB (39,901 cm^−1^) corresponds to the observed one (35,751 cm^−1^). The band origin of DMB is shown by an arrow at 35751 cm^−1^. Figure adapted from Reference [29].

The LIF spectra are compared to the transition energies of the conformers I–VIII (red bars) obtained by TDDFT calculations in Figure 12. The calculated transition energies are scaled by a factor of 0.89599 so that the calculated transition energy of DMB (39,901 cm^−1^) fits to the observed value (35,751 cm^−1^ [47], indicated by an arrow). Since the bands **M1–M4** are located in the lower-frequency side of the DMB origin band, the conformer IV can be excluded as a candidate assignable to species **M1–M4**. Moreover, in the geometry optimization of B18C6-(H_2_O)_1_, we found that conformers II, V, and VII cannot incorporate a H_2_O molecule in a bidentate H-bonding manner.

In the experimental studies the addition of water vapor reduces the intensities of the **M2–M4** bands, suggesting that species **M2–M4** can effectively incorporate H_2_O molecule(s). Thus the conformers II, V, and VII can also be excluded as candidates for species **M1–M4**. Among the remaining conformers (I, III, VI, and VIII), conformer VIII has a much lower transition energy so we assign species **M1** to conformer VIII. Conformers I, III, and VI can be assigned to species **M4**, **M2** and **M3**, respectively, from the relative positions of the transition energies albeit there is a large uncertainty on the assignment. Table 3 collects the positions of the band origin, the size of the species and their structural assignment.

#### 3.2.4. Structure of B18C6-(H_2_O)_1_

**Figure 13.**
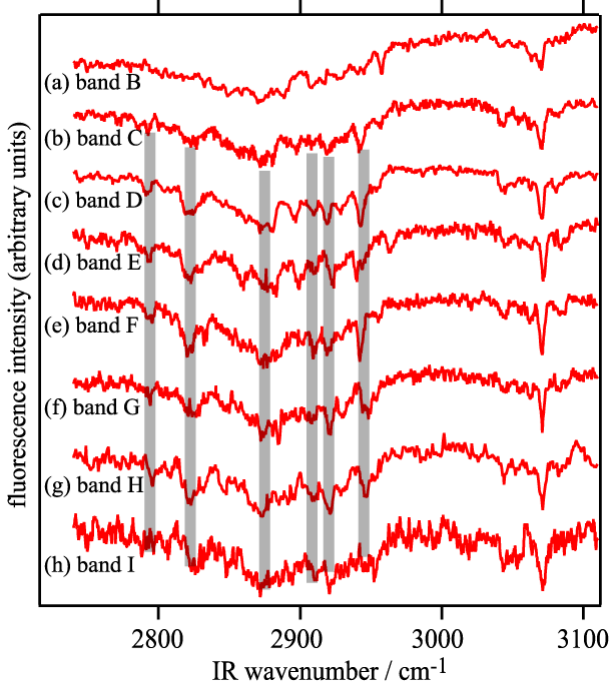
IR-UV DR spectra of bands B-I in the CH stretching region for B18C6-(H_2_O)*_n_*. Thick lines highlight similar features in the spectra of bands C–I at ∼2790, ∼2820, ∼2870, ∼2910, ∼2920, and ∼2940 cm^−1^. Figure adapted from Reference [29].

Although a brief description of the H-bonding networks present in B18C6-(H_2_O)*_n_* was given based on the IR-UV DR spectra in the OH stretching region, the conformation of the crown part has not been assigned yet. In this section we therefore discuss the more detailed structures of B18C6-(H_2_O)*_n_* including the conformation of the host B18C6. Figure 13 displays the IR-UV DR spectra of B18C6-(H_2_O)*_n_* in the CH stretching region (species **B–I**). As seen in the figure, the IR spectra of species **C–I** exhibit similar features with each other as highlighted by the thick gray lines. This similarity strongly suggests that the species **C–I** have a similar conformation of the B18C6 host. On the contrary, the IR spectrum of species **B** is different from those of species **C–I**, suggesting that species **B** has a different B18C6 conformation from the others. As described above species **D** is the most predominant species in the B18C6-(H_2_O)_1_ isomers. The hydration process of B18C6 can be summarized as follows: a water molecule is selectively bound in a particular B18C6 conformation (production of species **D**) and further hydration occurs on this B18C6-(H_2_O)_1_ complex (production of species **E–I**). The result that only one conformer remains as a major species in the B18C6-(H_2_O)_n≥1_ complexes although there are four different conformers of bare B18C6 before the hydration is one of the most important findings for the B18C6/water system.

**Figure 14.**
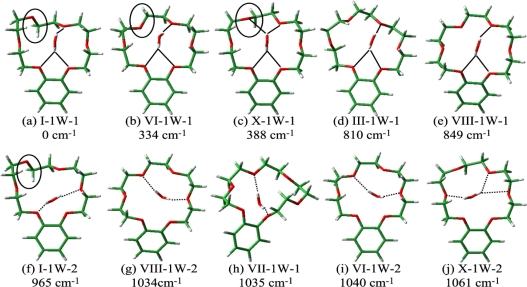
Optimized structures of B18C6-(H_2_O)_1_ at the B3LYP/6-31+G* level. The numbers shown in cm^−1^ represent the electronic energies of the isomers relative to that of the most stable isomer (I-1W-1). Solid and dotted lines denote H-bonding from the front and back sides of B18C6, respectively. Figure adapted from Reference [29].

The geometry optimization and vibrational analysis for B18C6-(H_2_O)_1_ was carried out at the B3LYP/6-31+G* level of theory starting with the initial geometries of conformers I–VIII (Figure 11) for the B18C6 host in the complex. Though more than 20 isomers are obtained, the 10 most stable ones are shown in Figure 14. The labeling of the isomers contains the information regarding the conformation of the B18C6 host and the number of H_2_O molecules. For example, isomer I-1W-1 [Figure 14(a)] has a B18C6 conformation similar to that of conformer I and encapsulates one H_2_O molecule (1W). The number at the end helps identify a specific isomer having the same B18C6 conformation and the same number of H_2_O molecules. It should be noted that isomer X-1W-1 [Figure 14(c)] has the B18C6 conformation similar to the conformers I and VI; the difference of the orientations of the O atoms are highlighted by the solid circles in Figure 14(a)–(c) and (f). However, the conformer X is obtained only in the presence of water molecules (*i.e.*, as a conformer of the bare B18C6).

As mentioned above the LIF and IR-UV DR spectra suggest two structural motifs for B18C6-(H_2_O)_1_: First, the H_2_O molecule should be H-bonded to the O atom(s) next to the benzene ring. Second, H_2_O is H-bonded in a bidentate fashion to the crown ring. Among the isomers shown in Figure 14, the six most stable isomers [Figures 14(a)–(f)] meet the two conditions so they are among the candidates for species **A–D**. It should be noted that in the five most stable isomers [Figure 14(a)–(e)], one of the OH groups of H_2_O is H-bonded to both O atoms (O_1_ and O_6_) adjacent to the benzene ring (bifurcated H-bond), similar to the case of DB18C6-(H_2_O)_1_. The calculated IR spectra in the OH stretching region for the 6 most stable B18C6-(H_2_O)_1_ isomers are displayed in the left side of Figure 9. As seen in Figure 9, all the calculated spectra are quite similar with each other, making it difficult to assign which spectra correspond to the observed IR spectrum of species **A–D**. We once again resort to the comparison of the electronic transition energies and the geometries in order to assign the species.

The calculated electronic transition energies for the six most stable B18C6-(H_2_O)_1_ isomers are indicated by blue bars in Figure 12. All B18C6-(H_2_O)_1_ isomers show blue shifts with respect to the corresponding bare molecular hosts because the water molecules in the isomers are H-bonded to the O atom(s) next to the benzene ring. Since species **A** is located on the higher-frequency side of the band **M1** (VIII), isomer VIII-1W-1 can be attributed to species **A**. As described above, species **C** and **D** have a similar B18C6 conformation with that of species **E–I** in larger complexes. Therefore species **C** and **D** should incorporate additional H_2_O molecules with no or a small change in the B18C6 conformation. Conformer I cannot incorporate two bidentate H_2_O molecules without changing its conformation, so that the isomers I-1W-1 and I-1W-2 can be excluded as candidates for species **C** and **D**. On the contrary, conformers VI and X can incorporate two bidentate water molecules. Therefore isomers VI-1W-1 and X-1W-1 can be assigned to species **C** and **D**. As for species **B**, we described that the conformation of the B18C6 host is quite different than that of species **C** and **D** as well as larger size complexes (species **E**–**I**). The isomer III-1W-1 fits to the condition that the conformation of III is quite different from that of VI and X. In addition, conformer III does not form stable larger size B18C6-(H_2_O)*_n_* (*n* > 1) complexes which reproduce the IR-UV DR spectra in the OH stretching region. Therefore isomer III-1W-1 can be assigned to species **B**.

We finally comment on the relative stability of the four species **A–D** of the B18C6-(H_2_O)_1_ isomer. In the experiment species **D** shows a much stronger LIF intensity than the other species **A–C**. Although this suggests a larger stability of species **D**, we could not obtain a specially stabilized isomer in the DFT calculations, that is, neither VI-1W-1 nor X-1W-1 is the most stable isomer. A possible explanation for this discrepancy can be possibly attributed to the inability of DFT to accurately reproduce the relative stability of the various isomers.

#### 3.2.5. Structures of B18C6-(H_2_O)_2–4_

**Figure 15.**
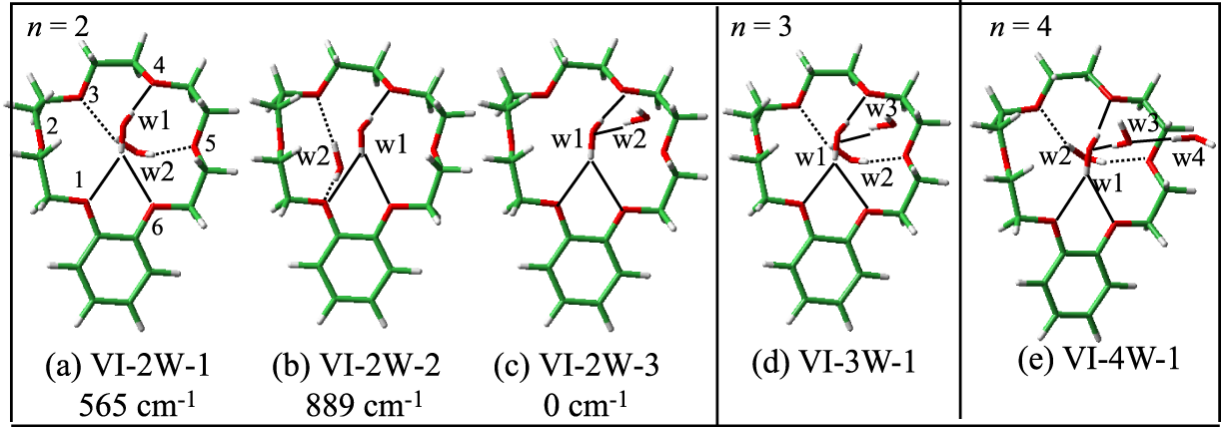
Optimized structures of (a–c) B18C6-(H_2_O)_2_, (d) B18C6-(H_2_O)_3_, and (e) B18C6-(H_2_O)_4_ at the B3LYP/6-31+G* level. The numbers shown in cm^−1^ represent the electronic energies of the isomers relative to that of VI-2W-3. Solid and dotted lines show H-bonding from the front and back sides of B18C6, respectively. Figure adapted from Reference [29].

The B18C6 part of the B18C6-(H_2_O)_2–4_ complexes should have the conformation of either the VI or X because the B18C6 conformation of species **E–I** is very similar to species **C** and **D**. We therefore carried out geometry optimizations for B18C6-(H_2_O)_2–4_ (species **E–I**) by adding H_2_O molecules to the conformers I, VI, and X as initial geometries. Figure 15 shows the optimized structures for B18C6-(H_2_O)_2–4_ formed by adding water to conformer VI. Although the conformation X also provides similar structures, the electronic energies of B18C6-(H_2_O)_2–4_ formed by adding water to the X conformer are higher by a few hundreds of cm^−1^ than those formed by adding water to conformer VI. As to conformer I, we found that this conformer cannot incorporate two H_2_O molecules in a bidentate manner different from conformer VI. This can be explained by the structural characteristics of VI-2W-1 [Figure 15(a)] and VI-2W-2 [Figure 15(b)] isomers. In these two complexes the second H_2_O molecule [referred to as (w2)] are H-bonded in a bidentate motif to O_3_ and O_5_ or to O_3_ and O_1_. In conformer I [Figure 11(a)], on the other hand, the O_3_ atom points out of the center of the crown ring. It is therefore difficult for conformer I to accept two bidentate H_2_O molecules. Following these considerations we assign the crown part in B18C6-(H_2_O)_2–4_ to conformation VI.

The calculated IR spectra for isomers VI-2W-1, VI-2W-2 and VI-2W-3 are shown in Figure 9. Isomers VI-2W-1 and VI-2W-2 exhibit different spectral features, albeit both isomers have two bidentate H_2_O molecules. In the case of isomer VI-2W-1 two pairs of the bidentate OH stretching vibrations are close to each other, whereas those of isomer VI-2W-2 are apart by ∼30 cm^−1^. Since these IR spectral features reproduce the IR-UV DR spectra of species **E** and **G** quite well, isomers VI-2W-1 and VI-2W-2 are assigned to species **E** and **G**, respectively. Furthermore the IR spectrum of VI-2W-3 also reproduces the IR-UV DR spectrum of band **F**. Therefore species **F** has the structure of isomer VI-2W-3. The optimized structures of B18C6-(H_2_O)_3,4_ are shown in Figure 15(d) and (e). Isomers VI-3W-1 and VI-4W-1 have the H-bond networks shown in Figure 10(d) and (e), respectively, and their IR spectra reproduce the IR-UV DR spectra of species **H** and **I** as seen in Figure 9. Thus isomers VI-3W-1 and VI-4W-1 are assigned to species **H** and **I**, respectively. The calculated S_1_−S_0_ transition energies of the isomers shown in Figure 15 are displayed in Figure 12(g) as green bars. As the number of H_2_O molecules increases, the transition energy is more blue-shifted. This trend in the calculated transition energies is consistent with the experimental results obtained from the LIF spectra. All isomers of the B18C6-(H_2_O)_2–4_ complexes shown in Figure 15 have one H_2_O molecule (w1) H-bonded to the O atoms adjacent to the benzene ring in a bidentate and bifurcated fashion. Further hydration networks are extended on the structure of isomer VI-1W-1; the first encapsulated water molecule (w1) in VI-1W-1 plays a role in the “nucleation” of the hydrated complexes.

### 3.3. Calix[4]arene (C4A)

#### 3.3.1. Electronic Spectra of C4A and C4A 6-(H_2_O)_1_

**Figure 16.**
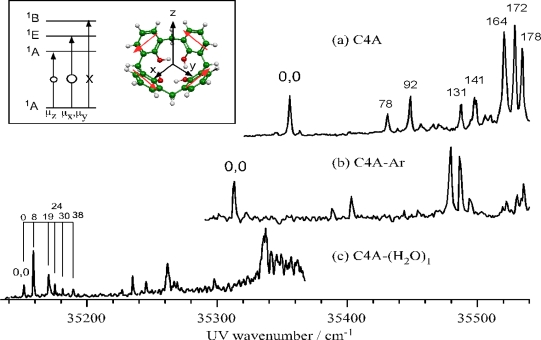
Mass resolved two-color REMPI spectra of (a) C4A, (b) C4A-Ar complex and (c) C4A-(H_2_O)_1_ complex in supersonic beams. Figure adapted from Reference [33].

Figure 16(a) shows the mass-resolved two color REMPI spectra of jet-cooled C4A. The electronic structure of C4A is expressed by the linear combination of that of four phenol molecules. The electronic states of C4A are split into the ^1^A, ^1^E and ^1^B states under the *C*_4_ point group. Among them the ^1^A and ^1^E states are dipole allowed from the ground state. However, if the transition moment of each phenol is in the (*x*,*y*)-plane (see the inset of Figure 16), the probability of the ^1^A←S_0_(^1^A) transition becomes zero and only the ^1^E←S_0_(^1^A) transition has a non-zero intensity. Consequently, the ^1^A←S_0_(^1^A) transition will have non-zero intensity if the transition moment of each phenol moiety is tilted from the (*x*,*y*)-plane. In the obtained spectrum the band at 35,357 cm^−1^ is assigned to the band origin of the S_1_(^1^A)←S_0_(^1^A) transition. In the higher frequency region there are four bands up to 141 cm^−1^ and three intense bands at ∼170 cm^−1^. The vibronic bands higher than this region (not shown here) can be assigned to combination bands.

Figures 16(b) and (c) show the mass-resolved two-color REMPI spectra of C4A-Ar and C4A-(H_2_O)_1_ complexes, respectively. The vibronic pattern of C4A-Ar is very similar to that of the bare C4A, although all bands are red-shifted by 45 cm^−1^ with respect to those of C4A. The C4A-Ar complex is an *endo*-complex, *i.e.*, one in which the Ar atom is located along the C_4_ axis inside the C4A cavity [54]. In contrast, the spectrum of C4A-(H_2_O)_1_ is quite different from that of either C4A or C4A-Ar. The weak origin band at 35,151 cm^−1^ is red-shifted by 206 cm^−1^ from the one of bare C4A. Several low frequency vibronic bands emerge up to ∼40 cm^−1^ above the origin, which can be attributed to the intermolecular modes such as the internal rotation of the water molecule. The strong vibronic bands at 0,0 + 190 cm^−1^ of C4A-(H_2_O)_1_ become broader than the corresponding bands of bare C4A.

#### 3.3.2. IR-UV DR and IRPD Spectra

**Figure 17.**
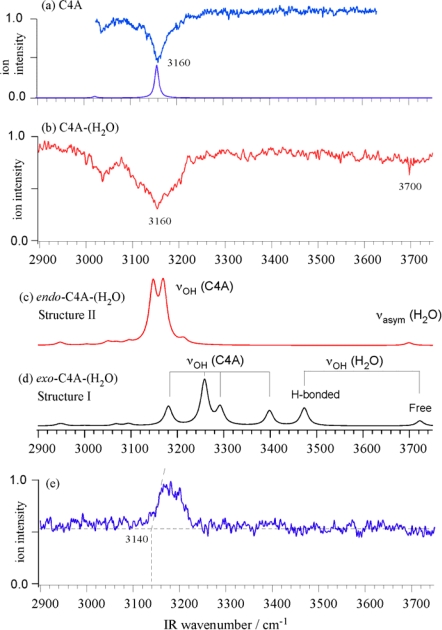
IR-UV dip spectra of (a) C4A and (b) C4A-(H_2_O). IR spectra of (c) *endo*-(Structure II) and (d) *exo*-form of C4A-(H_2_O) (Structure I) obtained at the MP2/aug-cc-pVDZ level of theory. The calculated frequencies are scaled by 0.96. (e) IRPD spectrum of C4A-(H_2_O). Figure adapted from Reference [33].

Figures 17(a) and (b) show the IR-UV DR spectra of the bare C4A the C4A-(H_2_O)_1_ complex, respectively. The IR-UV DR spectrum of C4A exhibits a strong and broad OH stretching band centered at 3160 cm^−1^. This band is red-shifted by ∼500 cm^−1^ from the frequency of the free OH stretch of phenol (3657 cm^−1^). The weak band at 3040 cm^−1^ is assigned to the CH stretching vibration of the aromatic ring. In the IR-UV DR spectrum of the C4A-(H_2_O)_1_ complex this H-bonded OH stretch band also emerges at the same frequency as that for bare C4A but its bandwidth is wider, which may be due to the overlap of several bands. The fact that the OH stretching frequency of C4A-(H_2_O)_1_ is the same with that of bare C4A indicates that the OH groups of the C4A moiety are not affected by the complexation with H_2_O, that is, the water molecule is not bound to the OH groups of C4A. In addition to the strong band at 3160 cm^−1^, the IR-UV DR spectrum exhibits a weak band at 3700 cm^−1^, which is assigned to the anti-symmetric or free OH stretching vibration of the water molecule. Figure 17(e) shows the IRPD spectrum of C4A-(H_2_O)_1_. The spectrum is obtained by scanning the IR laser frequency while monitoring the C4A^+^ signal with a UV frequency fixed at 10 cm^−1^ lower frequency side of the band origin of C4A. When we compare the IRPD and IR-UV DR spectra we can see a sharp cutoff at 3140 ± 20 cm^−1^ in the IRPD spectrum and the C4A^+^ fragment is not detected below this frequency, although the C4A-(H_2_O)_1_ complex shows the IR absorption in the IR-UV DR spectrum. Therefore this threshold (3140 ± 20 cm^−1^) corresponds to the C4A-(H_2_O)_1_ → C4A + H_2_O dissociation energy. We note that it is accidental that the IR absorption band coincided with the binding energy of the complex.

#### 3.3.3. Structure of C4A-(H_2_O)_1_

**Figure 18.**
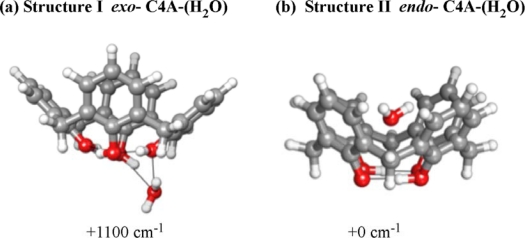
(a) Optimized structures of the *exo*- (Structure I) and *endo*-isomers (Structure II) of the C4A-(H_2_O)_1_ complex obtained at the MP2/aug-cc-pVDZ level of theory. The numbers shown in cm^−1^ are electronic energies relative to that of endo-isomer. Figure adapted from Reference [33].

In order to obtain the optimal structures of the C4A-(H_2_O)_1_ complex, we performed geometry optimizations at the B3LYP/6-31+G** and MP2/aug-cc-pVDZ levels of theory. The former calculation yields four *exo*-conformers (not shown here), but failed to yield a stable *endo*-isomer structure. In contrast, the MP2/aug-cc-pVDZ optimizations did produce a stable *endo*-isomer structure, which was determined to be the global minimum (*vide infra*). The minimum energy structures of the most stable among the *exo*-conformers (Structure I) and the global minimum *endo*-isomer (Structure II) of the C4A-(H_2_O)_1_ complex obtained at the MP2/aug-cc-pVDZ level of theory are shown in Figure 18.

The relative energies, *ΔE*_endo_ and *ΔE*_exo_, of the *endo*- and *exo*-isomers (in cm^−1^) and their separation, *ΔΔE* = (*ΔE*_endo_-*ΔE*_exo_) are listed in Table 4 at the MP2 level of theory with the aug-cc-pV*n*Z, *n* = D, T, Q basis sets together with the relaxation energies for the C4A and water (obtained from equations 3(a) and 3(b)). These are the energy penalties for distorting the geometries of the two moieties (C4A and water) from their gas phase structures to the ones they assume in the C4A-(H_2_O)_1_ complex. The BSSE-corrected isomer binding energies (calculated from equation (2)) are also shown in parentheses. We note that the *endo*-isomer is always more stable (with any of the aug-cc-pV*n*Z, *n* = D, T, Q basis sets) than the *exo*-isomer. Furthermore, the energy separation between the two isomers converges with basis set to about 1,100 cm^−1^, the *endo*-isomer being more stable. As expected from the optimal geometries shown in Figure 18, the C4A moiety is much more distorted in the *exo-* than in the *endo*-isomers, whereas the distortion of the water molecule in both isomers is minimal, as indicated from the magnitude of the relaxation energies for the two moieties listed in Table 4. Finally the best computed (MP2/aug-cc-pVQZ) binding energy of 3127 cm^−1^ of the most stable *endo*-isomer is in excellent agreement with the experimentally determined threshold of 3140 ± 20 cm^−1^. The variation of the uncorrected and BSSE-corrected binding energies listed in Table 4 suggests that the MP2/aug-cc-pVQZ energy is probably an upper limit for the binding energy of the *endo*-isomer.

In the *exo*-isomer (Structure I) the water molecule is inserted into and enlarges the ring homodromic network originally formed by the four OH groups of C4A. The resulting five OH homodromic ring is consistent with the network having the largest cooperativity [55]. In this structure, the H-bonding network of the OH groups in C4A is largely distorted by the insertion of the water molecule by ca. 2,000 cm^−1^ as reported in Table 4. As a result, in the calculated IR spectrum of the *exo*-isomer (Structure I) the degenerate OH stretching bands in the C4A moiety are split as indicated in Figure 17(d).

In contrast, the IR spectrum of the most stable *endo*-isomer (Structure II) is much more simpler, attesting to the minimal (∼100 cm^−1^) distortion of the C4A moiety in the C4A-(H_2_O)_1_ complex. In the observed IR spectrum of the *endo*-isomer, there is no band between 3,250 and 3,650 cm^−1^. This spectral pattern is consistent with the theoretically calculated IR spectrum at the MP2/aug-cc-pVDZ level of theory. The calculated IR spectrum of “Structure II” [Figure 17(c)] shows a perfect agreement with the observed one [Figure 17 (b)]. The degenerate OH stretching bands of C4A at 3,160 cm^−1^ are split into two due to the symmetry reduction since the encapsulation of the water molecule lowers the symmetry of C4A. This is the reason why the observed band at 3160 cm^−1^ is broader than that of bare C4A. The band at 3,700 cm^−1^ is assigned to the anti-symmetric OH stretching vibration (ν_3_) of the encapsulated water. In the *endo*-isomer (Structure II) the two OH groups of the water molecule are bound to two phenyl rings in a bidentate manner and the oxygen atom of the water molecule is facing towards the rim of C4A. The reason for this arrangement can be thought of originating from the dipole-dipole interactions between C4A and the water molecule. The dipole moment of C4A is 2.37 Debye, oriented along the C_4_ axis and pointing in a downward direction towards the rim. Thus, the oxygen atom of the water molecule in the cavity prefers to be oriented towards the rim of C4A in order to maximize the dipole-dipole interaction between the two fragments. It should be noted that the encapsulated water molecule retains the IR activity similar to the gas phase water, that is the IR intensity of ν_3_ is much stronger than ν_1_, though the ν_3_ frequency is 56 cm^−1^ lower than that of water in the gas phase (3755.8 cm^−1^)[46]. This lower frequency shift indicates both the OH groups of the water in the cavity are bound to the phenyl group by the OH-π H-bond. For comparison, the OH stretching vibration of the water molecule with the OH-π H-bonding occurs at 3,636–3,657 cm^−1^ in the benzene-(H_2_O)_n_ complexes [56]. The smaller red-shift of the OH stretching frequency in the C4A-(H_2_O)_1_ than the benzene-(H_2_O)_n_ complex is attributed to that the orientation of the water molecule in structure II is not favored for the most stable OH-π formation and to the splitting of the two equivalent OH oscillators in the water. In any case, the synergy between the two OH-π H-bonding and dipole-dipole interactions results in the large stabilization energy of “Structure II” with respect to the H-bonded homodromic *exo*-isomer (Structure I).

The combination of the above findings, namely that (1) the *endo*-isomer is lower in energy at the MP2 level of theory, (2) its best estimate for the binding energy matches within 15 cm^−1^ the experimentally determined threshold for the dissociation energy of the complex into the C4A and water fragments and (3) the computed IR spectrum of the *endo*-isomer shows a perfect agreement with the experimentally observed one, makes the unambiguous assignment that the observed structure corresponds to the *endo*-isomer of the C4A-(H_2_O)_1_ complex, in which the water molecule resides within the C4A cavity.

## 4. Conclusions

Water containing complexes of DB18C6, B18C6, and C4A were generated in supersonic jets and their structures determined by various laser spectroscopic methods with the aid of quantum chemical calculations. The overall structural motifs of the various complexes are determined by balancing several interactions. Among them, the OH---O hydrogen bonding is most important for DB18C6 and B18C6, while the dipole-dipole and OH---π hydrogen bonding are important for C4A. In addition to these interactions, we found that the conformation and flexibility are important factors to yield stable water-containing complexes of B18C6. In the supersonic jets, we identified four conformers for bare B18C6, but only one conformer (conformer VI) remained as a suitable choice for encapsulating water molecule(s) in its cavity. For the C4A-(H_2_O)_1_ complex the most stable conformer is the *endo*-isomer in which the water molecule is encapsulated inside the cavity of C4A. The fact that the *endo*-isomer is more stable than *exo*-isomer was surprising for us, since we initially expected opposite stability from the knowledge of behavior of C4A in condensed phase. This bonding arrangement offers the possibility to probe the delicate balance between the cumulative OH-π H-bonding and dipole-dipole interactions on one hand and the maximization of the cooperative effects associated with the formation of H-bonded homodromic networks on the other. Since the B3LYP/6-31+G** level of theory failed to produce a stable *endo*-isomer, in contrast to MP2, higher levels of electron correlation will be required in order to study functional molecules in which both the hydrophilic and hydrophobic interactions are important. Although the laser spectroscopic studies on the functional molecules in the gas phase have been initiated only recently, our knowledge on the encapsulation structure, solvent effect, conformer selectivity and major host–guest interaction in molecular level is increasing very rapidly. The information will be useful for designing host-guest systems to form specific encapsulation structure in condensed phase. To this end, work in the near future will be extended to much larger functional molecules which can incorporate various ion and neutral guest agents in micro-solvated conditions.

## Figures and Tables

**Table 1.** Origin, positions, size and structural assignment for DB18C6 and its hydrated complexes.

**Position / cm^−1^**	**Label**	**Size**	**Assignment**
35,597	**m1**	DB18C6	Chair-I
35,688	**m2**		Boat
35,777	**a**	DB18C6-(H_2_O)_1_	1W-1
35,800	**b**		1W-2
35,835	**c**	DB18C6-(H_2_O)_2_	2W-1
35,858	**f**	DB18C6-(H_2_O)_4_	4W-2
35,912	**d**	DB18C6-(H_2_O)_3_	3W-1
35,955	**e**	DB18C6-(H_2_O)_4_	4W-1

**Table 2.** S_1_-S_0_ and S_2_-S_0_ transition energies and oscillator strengths of DB18C6. The calculated values are obtained at the TD-B3LYP/6-31+G* level without scaling.

	Transition energy / cm^−1^				Oscillator strength	
	Obs.		Calc.		Calc.	
	S_1_-S_0_	S_2_-S_0_	S_1_-S_0_	S_2_-S_0_	S_1_-S_0_	S_2_-S_0_
Boat (*C*_2v_)	35,688	35,693	39,640	39,722	0.0928	0.0347
Chair I (*C*_i_)	35,597		39,608	39,688	0.1382	0
Chair II (*C*_i_)			40,133	40,145	0.0963	0

**Table 3.** Origin, positions, size and structural assignment for B18C6 and its hydrated complexes.

Position / cm^−1^	Label	Size	Assignment
35,167	M1	B18C6	VIII
35,628	M2		III
35,659	M3		I or VI
35,666	M4		I or VI
35,253	A	DB18C6-(H_2_O)_1_	VIII-1W-1
35,758	B		III-1W-1
35,771	C		VI-1W-1 or X-1W-1
35,766	D		VI-1W-1 or X-1W-1
35,813	E	DB18C6-(H_2_O)_2_	VI-2W-1
35,825	F		VI-2W-3
35,827	G		VI-2W-2
35,837	H	DB18C6-(H_2_O)_3_	VI-3W-1
35,858	I	DB18C6-(H_2_O)_4_	VI-4W-1

**Table 4.** Total binding (*ΔE*_endo_, *ΔE*_exo_) and relative (*ΔΔE* = *ΔE*_endo_-*ΔE*_exo_) energies (in cm^−1^) of the global minimum *endo*- and the local minimum *exo*-isomers of the C4A-(H_2_O)_1_ complex. Numbers in parentheses indicate BSSE-corrected numbers. The relaxation energies for C4A and water (distortion energy of the C4A and water moieties in the complex from their gas phase structures) are also denoted for the two isomers.

Level of theory	*Endo*-form *ΔE*_endo_ / cm^−1^	*E*_relax_ / cm^−1^	*Exo*-form *ΔE*_exo_, cm^−1^	*E*_relax_ / cm^−1^	*ΔΔE* / cm^−1^
MP2/aug-cc-pVDZ	4,312 (2,455)	121 / 11	2,561 (1,349)	2,009 / 57	1,751 (1,106)
MP2/aug-cc-pVTZ	3,602 (2,789)	81 / 34	2,228 (1,687)	2,004 / 86	1,374 (1,102)
MP2/aug-cc-pVQZ	3,127		1,985		1,142

## References

[1] Gokel G.W. (1991). Crown Ethers and Cryptands.

[2] Dobler M. (1981). Ionophores and their Structures.

[3] Gutsche C.D., Stoddart J.F. (1989). “Calixarenes”. Monographs in Supramolecular Chemistry.

[4] Cram D.J., Cram J.M., Stoddart J.F. (1994). “Container molecules and their guests”. Monographss in Supramolecular Chemistry.

[5] Gutsche C.D., Stoddart J.F. (1998). “Calixarenes revisited”. Monographs in Supramolecular Chemistry.

[6] Stuart A.M., Vidal J.A. (2007). Perfluoroalkylated 4,13-diaza-18-crown-6 ethers: synthesis, phase-transfer catalysis, and recycling studies. J. Org. Chem.

[7] Chekholov A.N. (2008). (18-crown-6)potassium tris(thiocyanato)nickelate(II): Synthesis and crystal structure. Russ. J. Coord. Chem.

[8] Atwood J.L., Koutsoantonis G.A., Raston C.L. (1994). Purification of C_60_ and C_70_ by selective complexation with calixarenes. Nature.

[9] Suzuki T., Nakashima K., Shinkai S. (1994). Very Convenient and efficient purification method for fullerene (C_60_) with 5,11,17,23,29,35,41,47-Octa-*tert*-butylcalix[8]arene-49,50,51,52,53,54,55,56-octol. Chem. Lett.

[10] Izatt R.M., Rytting J.H., Nelson D.P., Haymore B.L., Christensen J.J. (1969). Binding of alkali metal ions by cyclic polyethers: significance in ion transport processes. Science.

[11] Izatt R.M., Nelson D.P., Rytting J.H., Haymore B.L., Christensen J.J. (1971). Calorimetric study of the interaction in aqueous solution of several uni- and bivalent metal ions with the cyclic polyether dicyclohexyl-18-crown-6 at 10,25, and 40.deg. J. Am. Chem. Soc.

[12] Izatt R.M., Terry R.E., Haymore B.L., Hansen L.D., Dalley N.K., Avondet A.G., Christensen J.J. (1976). Calorimetric titration study of the interaction of several uni- and bivalent cations with 15-crown-5, 18-crown-6, and two isomers of dicyclohexo-18-crown-6 in aqueous solution at 25.degree.C and .mu. = 0.1. J. Am. Chem. Soc.

[13] Izatt R.M., Terry R.E., Nelson D.P., Chan Y., Eatough D.J., Bradshaw J.S., Hansen L.D., Christensen J.J. (1976). Calorimetric titration study of the interaction of some uni- and bivalent cations with benzo-15-crown-5, 18-crown-6, dibenzo-24-crown-8, and dibenzo-27-crown-9 in methanol-water solvents, at 25.degree.C and .mu. = 0.1. J. Am. Chem. Soc.

[14] Lamb J.D., Izatt R.M., Swain C.S., Christensen J.J. (1980). A systematic study of the effect of macrocycle ring size and donor atom type on the log K, .DELTA.H, and T.DELTA.S of reactions at 25.degree.C in methanol of mono- and divalent cations with crown ethers. J. Am. Chem. Soc..

[15] Pedersen C.J., Frensdorff H.K. (1972). Macrocyclic polyethers and their complexes. Angew. Chem. Int. Ed.

[16] Glendening E.D., Feller D., Thompson M.A. (1994). An Ab Initio Investigation of the Structure and Alkali Metal Cation Selectivity of 18-Crown-6. J. Am. Chem. Soc.

[17] More M.B., Ray D., Armentrout P.D. (1999). Intrinsic affinities of alkali cations for 15-crown-5 and 18-crown-6: bond dissociation energies of gas-phase M^+^−crown ether complexes. J. Am. Chem. Soc.

[18] Hill S.E., Feller D. (2000). Theoretical study of cation/ether complexes: 15-crown-5 and its alkali metal complexes. Int. J. Mass Spectrom.

[19] Peiris D.M., Yang Y., Ramanathan R., Williams K.R., Watson C., Eyler J.R. (1996). Infrared multiphoton dissociation of electrosprayed crown ether complexes. Int. J. Mass Spectrom. Ion Process.

[20] Anderson J.D., Paulsen E.S., Dearden D. (2003). Alkali metal binding energies of dibenzo-18-crown-6: experimental and computational results. Int. J. Mass Spectrom.

[21] Armentrout P.B. (1999). Cation–ether complexes in the gas phase: thermodynamic insight into molecular recognition. Int. J. Mass Spectrom.

[22] Feller D. (1997). *Ab Initio* Study of M^+^:18-Crown-6 Microsolvation. J. Phys. Chem. A.

[23] Rodriguez J.D., Lisy J.M. (2009). Infrared spectroscopy of gas-phase hydrated K^+^:18-crown-6 complexes: Evidence for high energy conformer trapping using the argon tagging method. Int. J. Mass. spectrom.

[24] Rodriguez J.D., Lisy J.M. (2009). Infrared spectroscopy of multiply charged metal ions: methanol-solvated divalent manganese 18-crown-6 ether systems. J. Phys. Chem. A.

[25] Rodriguez J.D., Vaden T.D., Lisy J.M. (2009). Infrared spectroscopy of ionophore-model systems: hydrated alkali metal ion 18-crown-6 ether complexes. J. Am. Chem. Soc.

[26] Shubert V.A., Zwier T.S. (2007). IR-IR-UV Hole-burning: conformation specific IR spectra in the face of UV spectral overlap. J. Phys. Chem. A.

[27] Shubert V.A., James W.H., Zwier T.S. (2009). jet-cooled electronic and vibrational spectroscopy of crown ethers: benzo-15-crown-5 ether and 4′ -amino-benzo-15-crown-5 ether. J. Phys. Chem. A.

[28] Shubert V.A., Müller C.W., Zwier T.S. (2009). Water’s role in reshaping a macrocycle’s binding pocket: infrared and ultraviolet spectroscopy of Benzo-15-crown-5-(H_2_O)n and 4′-aminobenzo-15-crown-5-(H_2_O)*_n_*, *n* = 1, 2. J. Phys. Chem. A.

[29] Kusaka R., Inokuchi Y., Ebata T. (2007). Laser spectroscopic study on the conformations and the hydrated structures of benzo-18-crown-6-ether and dibenzo-18-crown-6-ether in supersonic jets. Phys. Chem. Chem. Phys.

[30] Kusaka R., Inokuchi Y., Ebata T. (2008). Structure of hydrated clusters of dibenzo-18-crown-6-ether in a supersonic jet—encapsulation of water molecules in the crown cavity. Phys. Chem. Chem. Phys.

[31] Kusaka R., Inokuchi Y., Ebata T. (2009). Water-mediated conformer optimization in benzo-18-crown-6-ether/water system. Phys. Chem. Chem. Phys.

[32] Ebata T., Hodono Y., Ito T., Inokuchi Y. (2007). Electronic spectra of jet-cooled calix[4]arene and its van der Waals clusters: Encapsulation of a neutral atom in a molecular bowl. J. Chem. Phys.

[33] Hontama N., Inokuchi Y., Ebata T., Dedonder-Lardeux C., Jouvet C., Xantheas S.S. (2010). Structure of the Calix[4]arene-(H_2_O) cluster: The world’s smallest cup of water. J. Phys. Chem. A.

[34] Ebata T. (2009). Study on the structure and vibrational dynamics of functional molecules and molecular clusters by double resonance vibrational spectroscopy. Bull. Chem. Soc. Jpn.

[35] Frisch M.J., Trucks G.W., Schlegel H.B., Scuseria G.E., Robb M.A., Cheesman J.R., Montgomery J.A., Vreven T., Kudin K.N., Burant J.C., Millam J.M., Iyengar S.S., Tomasi J., Barone V., Munnucci B., Cossi M., Scalmani G., Rega N., Petersson G.A., Nakatsuji H., Hada M., Ehara M., Toyota K., Fukuda R., Hasegawa J., Ishida M., Nakajima T., Honda Y., Kitao O., Nakai H., Klene M., Li X., Knox J.E., Hratchian H.P., Cross J.B., Bakken V., Asamo C., Jaramillo J., Gomperts R., Stratmann R.E., Yazyev O., Ausitin A.J., Cammi R., Pomelli C., Ochterski J.W., Ayala P.Y., Morokuma K., Voth G.A., Salvador P., Dannenberg J.J., Zakrzewski V.G., Dapprich S., Daniels A.D., Strain M.C., Farkas O., Malick D.K., Rabuck A.D., Raghavachari K., Foresman J.B., Ortiz J.V., Cui Q., Baboul A.G., Clifford S., Cioslowski J., Stefanov B.B., Liu G., Liashenko A., Piskorz P., Komaromi I., Martin R.L., Fox D.J., Keith T., Al-Laham M.A., Peng C.Y., Nanayakkara A., Challacombe M., Gill P.M. W., Johnson B., Chen W., Wong M.W., Conzalez C., Pople J.A. (2004). Gaussian 03.

[36] Møller C., Plesset M.S. (1934). Note on an approximation treatment for many-electron systems. Phys. Rev.

[37] Dunning T.H. (1989). Gaussian basis sets for use in correlated molecular calculations. I. The atoms boron through neon and hydrogen. J. Chem. Phys.

[38] Kendall R.A., Dunning T.H., Harrison R.J. (1992). Electron affinities of the first-row atoms revisited. Systematic basis sets and wave functions. J. Chem. Phys.

[39] Kendall R.A., Aprà E., Bernholdt D.E., Bylaska E.J., Dupuis M., Fann G.I., Harrison R.J., Ju J., Nichols J.A., Nieplocha J., Straatsma T.P., Windus T.L., Wong A.T. (2000). High performance computational chemistry: An overview of NWChem a distributed parallel application. Comp. Phys. Comm.

[40] Hehre W.J., Ditchfield R., Pople J.A. (1972). Self—consistent molecular orbital methods. XII. further extensions of gaussian—type basis sets for use in molecular orbital studies of organic molecules. J. Chem. Phys.

[41] Boys S.F., Bernardi F. (1970). The calculation of small molecular interactions by the differences of separate total energies. Some procedures with reduced errors. Mol. Phys.

[42] Xantheas S.S. (1996). On the importance of the fragment relaxation energy terms in the estimation of the basis set superposition error correction to the intermolecular interaction energy. J. Chem. Phys.

[43] Bright D., Truter M.R. (1970). Crystal structures of complexes between alkali-metal salts and cyclic polyethers. Part I. Complex formed between rubidium sodium isothiocyanate and 2,3,11,12-dibenzo-1,4,7,10,13,16-hexaoxocyclo-octadeca-2,11-diene (‘dibenzo-18-crown-6’). J. Chem. Soc. B.

[44] Müller A., Talbot F., Leutwyler S. (2002). S_1_/S_2_ exciton splitting in the (2-pyridone)_2_ dimer. J. Chem. Phys.

[45] Wessel J.E., Syage J.A. (1990). Excitonic interactions in naphthalene clusters. J. Phys. Chem.

[46] Herzberg G. (1945). Molecular Spectra And Molecular Structure Volume II. Infrared and Raman Spectra of Polyatomic Molecules.

[47] Yi J.Y., Ribblett J.W., Pratt D.W. (2005). Rotationally resolved electronic spectra of 1,2-dimethoxybenzene and the 1,2-dimethoxybenzene−water complex. J. Phys. Chem. A.

[48] Bühl M., Wipff G. (2002). Hydronium Ion complex of 18-crown-6: where are the protons? a density functional study of static and dynamic properties. J. Am. Chem. Soc.

[49] Goutev N., Matsuura H. (2001). Hydrogen Bonding in Chloroform Solutions of Ethylenedioxy Ethers. Spectroscopic Evidence of Bifurcated Hydrogen Bonds. J. Phys. Chem. A.

[50] Hang Z.S., Miller R.E. (1989). High-resolution near-infrared spectroscopy of water dimer. J. Chem. Phys.

[51] Fukuhara K., Tachikake M., Matsumoto S., Matsuura H. (1995). Raman spectroscopic study of the hydrates of 18-crown-6. J. Phys. Chem.

[52] Ebata T., Nagao K., Mikami N. (1998). Mode-dependent anharmonic coupling between OH stretching and intermolecular vibrations of the hydrogen-bonded clusters of phenol. Chem. Phys.

[53] Nibu Y., Marui R., Shimada H. (2006). Infrared spectroscopy of hydrogen-bonded 2-fluoropyridine−water clusters in supersonic jets. J. Phys. Chem. A.

[54] Ebata T., Hontama N., Inokuchi Y., Haino T., Xantheas S.S. Encapsulation of Ar*_n_* complexes by calix[4]arene: *endo- vs. exo*-complexes. Phys. Chem. Chem. Phys.

[55] Xantheas S.S. (2000). Cooperativity and hydrogen bonding network in water clusters. Chem. Phys.

[56] Pribble R.N., Zwier T.S. (1994). Size-specific infrared spectra of benzene-(H_2_O)*_n_* clusters (*n* = 1 through 7): evidence for noncyclic (H_2_O)*_n_* Structures. Science.

